# Correction to ‘The reproducibility of research and the misinterpretation of *p*-values'

**DOI:** 10.1098/rsos.180100

**Published:** 2018-03-07

**Authors:** David Colquhoun

*R. Soc. open sci.* **4**, 171085. (Published 6 December 2017). (doi:10.1098/rsos.171085)

An author name was given wrongly in reference [17] of http://rsos.royalsocietypublishing.org/content/4/12/171085.

The author's name should be Hooper R and not Holder R, as presently written.

The corrected reference in full is:

Hooper R. 2009 The Bayesian interpretation of a *p*-value depends only weakly on statistical power in realistic situations. *J. Clin. Epidemiol*. **62**, e1242–e1247. (doi:10.1016/j.jclinepi.2009.02.004)

