# Supplementary material for: The reproducibility of research and the misinterpretation of p-values
Source: R Soc Open Sci. 2017 Dec 6;4(12):171085. doi: 10.1098/rsos.171085 (PMC5750014; doi:10.1098/rsos.171085)
Supplement: files-paper-2 [file rsos171085supp1.zip › Notes-on-use-of-R-scripts.pdf]

Notes on use of R scripts that accompany *The Reproducibility Of Research And The Misinterpretation Of P Values*

David Colquhoun

How to run R scripts

If you have never used R before, here's a quick start.

First download R and install it (from http://www.r-project.org/)

Then download R Studio (from http://www.rstudio.com/),

Dorothy Bishop has provided download instructions, and a short introduction to R, at http://www.slideshare.net/deevybishop/learning

Create a directory/folder named something convenient like R-progs and copy the downloaded scripts into it.

Lines that begin with # are comments, not code. Some variable names start with 'my' (intended to avoid clashes with pre-defined variables, but isn't usually necessary)

All you have to do is to set the inputs, which are gathered together between #START INPUTS and #END OF INPUTS

Now you are ready to run the script. Hit ctrl-alt-r and the script will run. Alternatively highlight the whole script (ctrl-A) and click the run button (the run button runs the highlighted sections of the script).

Text files (*.txt) provide a permanent record of the output in most scripts. They appear in the same directory as the R scripts, by default. Some scripts generate graphs. Each graph appears in a separate window. Save the ones you want to keep manually'

Statistical considerations

The question which we ask in refs [1 - 3] is as follows. If you observe a "significant" P value after doing a single unbiased experiment, what is the probability that your result is a false positive?. "False positive risk" (FPR) is defined here as the probability that a result which is "significant" at a specified P value, is a false positive result. It is defined and explained in ref [3] The same thing was called "false discovery rate"in refs [1] and [2], and it was called "false positive rate" in earlier drafts of ref [3]. The notation in this field is a mess and it's important to check the definitions in each paper.

There are two different ways to calculate FPR. These are explained in detail in section 10 of ref [1], and, more carefully, in section 3 of ref [3]. They can be called the *p-equals* method, and the *p-less-than* method. The latter definition is used most frequently (eg by Ioannidis and by Wacholder), but the former method is more appropriate for answering our question. All programs give results that are calculated

with both methods. The results with the *p-equals* method, give higher false positive risk, for any given P value, than the other method (see Fig 2 in ref[3]), but they are the appropriate way to answer the question.

The calc-fpr web application

Experience has shown that the R scripts are downloaded far less often than the pdf of the papers. So, to make it easier to do calculations yourself, we have designed a web calculator, with help from Colin Longstaff and Brendan Halpin. At present, the best place to find it is at http://fpr-calculator.uksouth.cloudapp.azure.com:3838/

Soon a permanent url will be http://fpr-calc.ucl.ac.uk/

Click on the calculations tab, and choose which calculation to do by selecting one of the three radio buttons (top left). The input boxes that are appropriate for the calculation will appear. There are three variables, observed *P* value, FPR and the prior probability that the null hypothesis is false. The calculator will work out any one of these, given numbers for the other two. All three calculations require also the number of observations in each sample, and the effect size, expressed as a multiple of the standard deviation of the observations (default value 1.0) The default number per sample is 16 which gives a power of 0.78 for $P = 0.05$ and effect size = 1 -see ref [1] and [3] for more details.

Note that all that matters is the effect size expressed as a multiple of the standard deviation of the original observations (sometimes known as *Cohen's d*). The true mean of sample 1 is always 0 (null hypothesis), The true mean of sample 2 is set to the normalised effect size so the true standard deviation can always be set to 1, with no loss of generality.

A real life example

A study of transcranial electromagnetic stimulation, published In *Science* concluded that it "improved associative memory performance", P = 0.043. If we assume that the experiment had adequate power (the sample size of 8 suggests that might be optimistic) then, in order to achieve a false positive risk of 5% when we observe P = 0.043, we would have to assume a prior probability of 0.85 that the effect on memory was genuine (found from radio button 1). Most people would think it was less than convincing to present an analysis based on the assumption that you were almost certain (probability 0.85) to be right before you did the experiment.
Another way to express the strength of the evidence provided by P = 0.043 is to note that it makes the existence of a real effect only 3.3 times as likely as the existence of no effect (likelihood ratio). This would correspond to a minimum false positive risk of 23% if we were willing to assume that non-specific electrical zapping of the brain was as likely as not to improve memory (prior probability of a real effect was 0.5) (found via radio button 3).

The radio button 2 option shows that in the most optimistic case (prior = 0.5), you need to have P = 0.008 to achieve an FPR of 5 percent. [example from ref 3]

Notes

From ver 1.1 of the web app onwards, and in the current R scripts, the effect size (expressed as a multiple of the standard deviation of the observations) should be entered. The same results are found if the power is kept constant. For example effect size = 1 and n = 16 gives power = 0.78. For an effect size of 0.5 SD, n = 61 gives similar power and similar FPR etc. And for an effect size of 0.2 SD, a power of 0.78 requires n = 375, and again this gives similar FPR etc. See ref [4]. So choose n so that the calculated power matches that of your experiment.

From ver 1.3 onwards, the values of power that are printed out are calculated for P = 0.05 and the specified effect size (expressed as a multiple of the standard deviation of the observations). (In earlier versions they were calculated using the observed P value.)

There is a popular account of the logic involved in ref [6]. And ref [3] has, on pp 18-19, a response to the recent 72 author paper, Benjamin et al [7], on related topics

References

1. Colquhoun D.(2014) An investigation of the false discovery rate and the misinterpretation of p-values. Royal Society Open Science. 1(3):140216. doi: 10.1098/rsos.140216. Click for full text
2. Colquhoun D. False discovery rates: the movie Click for YouTube
3. Colquhoun D. (2017). The reproducibility of research and misinterpretation of P values.bioRxiv, May 31, 2017, doi: http://dx.doi.org/10.1101/144337 Click for full text
4, Colquhoun. D, (2015) Response to comment by Loiselle & Ramchandra (2015), Royal Society Open Science: DOI: 10.1098/rsos.150319 Click for full text
6. Colquhoun D. (2016). The problem with p-values. Aeon Magazine Click for full text
7. Benjamin, D. et al. (2017) Redefine Statistical Significance. PsyArXiv Preprints, July 22, 2017. Click for full text
